# Primary dengue haemorrhagic fever in patients from northeast of Brazil is associated with high levels of interferon-β during acute phase

**DOI:** 10.1590/0074-02760150453

**Published:** 2016-06

**Authors:** Renato Antônio dos Santos Oliveira, Mayara Marques Carneiro da Silva, Carlos Eduardo Calzavara-Silva, Ana Maria Silva, Marli Tenório Cordeiro, Patrícia Muniz Mendes Freire de Moura, Paulo Neves Baptista, Ernesto Torres de Azevedo Marques, Laura Helena Vega Gonzales Gil

**Affiliations:** 1 Fundação Oswaldo Cruz, Centro de Pesquisas Aggeu Magalhães, Departamento de Virologia, Recife, PE, Brasil; 2 Fundação Oswaldo Cruz, Centro de Pesquisas René Rachou, Departamento de Imunologia, Laboratório de Imunologia Celular e Molecular, Belo Horizonte, MG, Brasil; 3 University of Pittsburgh, Center for Vaccine Research, Department of Infectious Diseases and Microbiology, Pittsburgh, PA, US; 4 Universidade de Pernambuco, Instituto de Ciências Biológicas, Recife, PE, Brasil; 5 Universidade de Pernambuco, Faculdade de Ciências Médicas, Recife, PE, Brasil

**Keywords:** dengue fever, dengue haemorrhagic fever, type I interferon, innate immunity

## Abstract

Dengue is an acute febrile disease caused by the mosquito-borne dengue virus (DENV) that according to clinical manifestations can be classified as asymptomatic, mild or severe dengue. Severe dengue cases have been associated with an unbalanced immune response characterised by an over secretion of inflammatory cytokines. In the present study we measured type I interferon (IFN-I) transcript and circulating levels in primary and secondary DENV infected patients. We observed that dengue fever (DF) and dengue haemorrhagic fever (DHF) patients express IFN-I differently. While DF and DHF patients express interferon-α similarly (52,71 ± 7,40 and 49,05 ± 7,70, respectively), IFN- β were associated with primary DHF patients. On the other hand, secondary DHF patients were not able to secrete large amounts of IFN- β which in turn may have influenced the high-level of viraemia. Our results suggest that, in patients from our cohort, infection by DENV serotype 3 elicits an innate response characterised by higher levels of IFN- β in the DHF patients with primary infection, which could contribute to control infection evidenced by the low-level of viraemia in these patients. The present findings may contribute to shed light in the role of innate immune response in dengue pathogenesis.

Dengue is an acute febrile disease caused by a small single-stranded RNA virus with four antigenically distinct serotypes [dengue virus (DENV)-1 to 4]. DENV belongs to the genus Flavivirus of the *Flaviviridae* family and is transmitted to humans by infected *Aedes aegypti* mosquitoes (Ross 2010). DENV is circulating in more than 100 countries worldwide and causes 400 million new cases each year, of which only 100 million are symptomatic (Messina et al. 2015). DENV infection presents different clinical manifestations ranging from asymptomatic to severe. Symptomatic DENV infection is classified according to the severity of the disease in dengue fever (DF) or dengue haemorrhagic fever (DHF). DF is a self-limiting febrile illness, whereas DHF is a life-threatening condition characterised by an increased capillary permeability, thrombocytopenia and bleeding events that could lead to shock (Back & Lundkvist 2013). Alternatively, the WHO proposed a new classification system based on clinical and laboratorial parameters. This classification stratifies the dengue cases in dengue without warning signs, dengue with warning signs and severe dengue (WHO 2009). Different theories have tried to explain the pathogenesis of dengue infection (Martina et al. 2009), however the precise mechanism that triggers DHF remains poorly understood, this way an early identification of factors that could predispose the development of DHF would be of great clinical relevance. One of the hypotheses, the antibody-dependent enhancement (ADE), proposes that DHF could be a consequence of an unbalanced immune response mediated by non-neutralising antibodies (Halstead & O’Rourke 1977, Halstead 1988). This condition would be characterised by increased monocyte activation and secretion of chemical mediators and pro-inflammatory cytokines (Green & Rothman 2006, Kurane 2007). Differences in the cytokines (TNF-a, IFN-y, IL-6 IL-13) (Dong et al. 2007, Priyadarshini et al. 2010) and chemokines (Il-8, MIF, IP-10, MCP-1, MIP-1b) production (Chen et al. 2006, Lee et al. 2006,Fink et al. 2007, Assunção-Miranda et al. 2010), in culture supernatants and plasma samples from DF and DHF patients have been described. High serum levels of the pro-inflammatory cytokines IFN- y and TNF-a have been associated with disease severity (Nguyen et al. 2004), while Macrophage Inflammatory Protein-1b (MIP-1b) has been associated with a good prognosis (Bozza et al. 2008).

Mechanisms of innate immune response mediated by interferon (IFN) represent the most important pathways of host defense directed to hinder viral replication. The name interferon arose precisely because of its ability to interfere with viral replication. The IFN family consists of IFNs type I, II and III. Type I interferons (IFN-I) include 13 different IFN-α subtypes and one each of IFN-β, IFN-ϖ, IFN-ε and IFN-κ, they are the most important cytokines involved in the control of viral infection and can be secreted by most nucleated cells (Sen 2001, Sadler & Williams 2008), specially by plasmacytoid dendritic cells (Siegal et al. 1999). The type I IFN secreted by virus infected cells acts in an paracrine and autocrine manner, through the binding to surface receptors initiating a signaling cascade through the JAK/STAT pathway which ends up regulating the expression of interferon-induced genes (ISG). These genes encode proteins that promote an “antiviral” state in both infected and non-infected cells, which inhibits the virus life cycle, hampering its spreading (Samuel 2001). In order to become successful pathogens, viruses have developed different strategies to subvert the antiviral activity of type I IFN. Many viruses encode proteins that antagonise key steps of either type I IFN induction or signaling pathways (Jones et al. 2005, Umareddy et al. 2008, Ashour et al. 2009, Li et al. 2013, Wu et al. 2013). The importance of type I IFN for DENV control was evidenced by the IFN-β dependence of cultured hepatocytes to control virus replication (Liang et al. 2011).

Tang et al. (2010) described IFN-α and IFN-β levels in a cohort of hospitalised DENV-1 patients and IFN-α levels were more elevated than IFN-β which levels were similar to uninfected control (Tang et al. 2010). Maximum plasma levels of IFN-α were generally not seen and took place before to peak viraemia in DENV-3 infected children (Libraty et al. 2002), and more recently in an elegant study, Gandini et al. 2013 demonstrated that circulating plasma levels of IFN-α were higher in mild dengue compared with severe dengue or healthy controls (Gandini et al. 2013). Although the levels of IFN-α has been well described, little is known about IFN-β circulating levels during the acute phase of infection. This report describes for the first time differences of circulating IFN-β between DF and DHF patients during the acute phase of disease, up to five days after onset of symptoms. The differences on IFN-β levels observed could lead to altered immune response which may play an important role on dengue pathogenesis.

## MATERIALS AND METHODS

*Patients and samples* - Blood samples were obtained from a cohort of 104 acute febrile dengue patients admitted to different hospitals in the city of Recife (Pernambuco, Brazil), as already described (Cordeiro et al. 2007a, b). Briefly, blood samples were sequentially obtained from each patient on the day of admission (day 1) and at days 3, 5, 7, 15 and 30. Dengue cases were confirmed by DENV isolation and/or viral RNA detection by reverse transcriptase polymerase chain reaction (RT-PCR) and/or by positive serology for anti-dengue IgM ELISA. Subsequently, samples were clinically classified as DF or DHF according to 1997 WHO criteria (WHO 1997). Data about demographic characteristics, clinical features and biochemical and haematological laboratory tests were also collected while patients were hospitalised. Cytokine assays were carried out on 44 serum samples from DF patients and 33 serum samples from DHF patients. The presence of IgM and/or IgG antibodies specific to dengue virus was detected using a capture ELISA Kit, according to the manufacturer’s instructions (E-DEN01M and E-DEN01G, PanBio).

*Interferon mRNA levels by real-time RT-PCR* - Quantitative real-time PCR (qPCR) assays were performed with peripheral blood mononuclear cells (PBMC) samples collected and purified from clinically classified DHF and DF patients by density gradient technique (Ficoll-Paque™ Plus- GE Healthcare, Uppsala-SE). At different time point of infection as follows: (i) During the acute phase, up to five days after the onset of symptoms; (ii) During the nonviraemic but symptomatic phase, from six-10 days after the onset of symptoms; and (iii) During the convalescent phase, > 10 days after the onset of symptoms.

Genes were amplified and detected using TaqMan® gene expression assays (Applied BioSystems, cat. 4331182 - gene id: IFNα-1: Hs00855471_g1; IFNβ-1: Hs01077958_s1 and IFNy: Hs00989291_m1). Total RNA was extracted using the RNeasy Mini Kit (Qiagen) and treated with DNAse (Qiagen) following the manufacturer’s protocols. Total RNA (1 μg) was reverse transcribed to cDNA using a Super-Script III First-Strand Synthesis System (Invitrogen) and Random Hexamer Primers (Invitrogen) under the following reaction conditions: 50ºC for 30 min, 85ºC for 5 min and then incubation on ice. RNase H (2 U) (Invitrogen) was added and samples were incubated at 37ºC for 20 min. qPCR was performed using the ABI PRISM 7500 (Applied BioSystems). cDNA obtained from the total RNA from the patients described above was used. β-actin gene was used to normalise the gene data. Reactions were performed in triplicate and contained 2 μL of cDNA, 6.25 μM of each specific primer (Applied BioSystems), TaqMan Universal PCR Master Mix (Applied BioSystems) and water, in a final volume of 25 μL. Triplicates of non-template controls were included for each qPCR experiment. Cycle conditions were as follows: after initial periods of 2 min at 50ºC and 10 min at 95ºC, samples were cycled 40 times at 95ºC for 15 s and 60ºC for 1 min. The baseline and threshold for cycle threshold (Ct) calculations were set automatically using Sequence Detection Software, version 1.4 (Applied BioSystems). The efficiency of amplification (E) of each target molecule was calculated from the slope of the standard curve (plot of Ct vs. the negative log10 concentration of the target) derived from the slopes {E = [10(-1/Slope)]-1}. Once all assays met the amplification efficiency criteria of 100% ± 10% (Livak & Schmittgen 2001), the 2-∆∆Ct method was used for relative calculations (Applied Biosystems 2010, 20,^2012^, Livak & Schmittgen 2001).

*Measurements of type I IFN levels by ELISA* - The levels of IFN-I (α & β) in the serum/plasma of patients up to five days after onset of symptoms were evaluated by quantitative enzyme-linked immunosorbent assay (ELISA). The lower limits of detection were 1.0 pg/mL and 2.3 pg/mL for the human IFN-α (My Biosource, San Diego, CA) and IFN-β (Axxora, Farmingdale, NY) kits, respectively. Optical densities at 450nm were measured on an ELISA dedicated instrument and cytokine concentrations were obtained using a standard curve. All determinations were performed in duplicates and arithmetic averages were calculated.

*Real time RT-PCR assay for dengue* - Viraemia was quantified and expressed as the number of RNA copies per mL. Viral RNA was extracted from 140mL of each individual serum sample using the QIAamp Viral RNA Mini Kit/QIAmp MiniElute Vírus Spin Reagent (Qiagen, Valencia, CA). Reverse transcriptions were performed using random primers (hexamers), RNAse inhibitors (RNAse OUT 40 U/mL), Superscript III reverse transcriptase 200 U/mL and 5mL of purified RNA, according to the manufacturer’s specifications (Invitrogen). Quantification was performed by Real Time PCR with SYBR Green-based specific to DENV3 serotype kit for dengue (DSSS-P29), kindly provided by Dr Hen Phon-Too (National University of Singapore), able to detect down to a minimum of 10 RNA copies/mL. Each RT-PCR reaction included 32 mL of the DSSS kit mix with primers (0.2 mM), XtensaÒ PCR buffer (Bioworks, Singapore), MgCl_2_ (2.5 mM) and 0.5 mL Platinum hot start polymerase (5U/mL; Invitrogen), 2 mL reverse transcription mix and water, making a final volume of 50 mL. Primers were synthetised with NS4A sequence tags (forward primers with 5’GTCAGAA(C/G)ATGGCGGTAGG3’ and reverse primers with 5’ CTTTCCAATCCCTTTACCTGATAT3’). Amplification conditions were as follows: 2 min at 50ºC, 3 min at 95ºC, then 40 cycles of 30 s at 95ºC and 1 min at 60ºC. qPCR assays were performed in the ABI PRISM 7500 Sequence Detection System (Applied Biosystems, CA, USA). ABI PRISM software (version 1.4) was used to analyse qPCR products considering the melting temperature (dissociation curve), amplification curve and standard deviation between duplicates for quantification. The efficiency of amplification was calculated for each plate using the threshold cycle value (Ct) and the slope of the standard curve. Standard curves were obtained by amplification of the appropriate standards (10^7^ to 10^10^ copies). False positive results due to contaminations were ruled out by running no-template controls (NTC). Sera from health humans were used as negative controls. The local DENV-3 isolate propagated in*Aedes albopictus* C6/36 cells (10^6^ DENV RNA copies) was used as positive control. All assays were performed in duplicates.

*Statistical analysis* - Statistical analyses were performed using GraphPad Prism version 4.0a for Macintosh OS X (GraphPad Software, San Diego, CA). Real-time PCR data were analysed with multiple comparison Kruskal-Wallis test with Dunn’s correction. Fisher’s exact test was used to evaluate frequencies of positivity in symptoms. Differences in demographic information between DF and DHF patients were evaluated by nonparametric Mann-Whitney U test. Cytokines levels were analysed by one-way analysis of variance (ANOVA) for mean comparison for multiple groups with post hoc Bonferroni’s test, p values ≤ 0.05 were considered significant.

*Ethics* - The procedure followed was in accordance with the ethical standards of the committee on human experimentation of the Aggeu Magalhães Research Center as well as with Helsinki Declaration of 1975. A written informed consent was obtained from all subjects, and the protocol for this study was approved by Aggeu Magalhães Research Center committee under the number CEP: 11/11.

## RESULTS

*Clinical characteristics of DENV-infected patients* - 104 patients (46% men and 54% women) with acute dengue infection were included in the present study, of which 48 (46%) were classified as DF and 56 (54%) as DHF. The characteristics of studied population are summarised in Table. Age and gender of DF and DHF groups were similar with no significant difference between their medians.

**TABLE:** Demography, clinical and laboratorial characteristics of study cohort

Cohort characteristics^a^	Dengue fever		Dengue haemorrhagic fever		p
	
	Median (95% CI)^bt^	N	Median (95% CI)	N	
Age (years)	12 (16-25)	48	24.5 (22-32)	56	ns
Gender (M:F)		27:21		21:35	ns

IgG, + : -		37:11		41:15	ns

White Blood Cells (WBC) (10^3^/mm^3^)	5.0 (4.6-6.3)	46	3.8 (3.8-6.1)	42	0.1954
Platelets (10^3^/mm^3^)	164.5 (148.6-217.5)	48	74.5 (69.7-117.6)	42	**< 0.0001** ^**c**^
Total albumin (g/L)	4.5 (4.2-4.6)	26	3.6 (3.4-4.0)	21	**0.0001** ^**c**^
Aspartate Transaminase (AST) (IU/L)	40.6 (51.2-124.2)	34	70 (58.7-157.3)	23	**0.0293** ^**c**^
Alanine Transaminase (ALT) (IU/L)	28.2 (25.62-101.3)	34	63 (54.9-119.7)	23	**0.0047** ^**c**^

Abdominal pain		30 (62.5%)^d^		31 (58.5%)	0.5501^f^
Hypotension		1 (2%)		7 (12.5%)	0.0662^f^
Bleeding manifestation^e^		6 (12.8%)		18 (32.1%)	**0.0206** ^**f**^

M: male; F: female; ns: not significant; ^a^study population with 104 patients; ^b^CI, 95% confidence interval;^c^Mann-Whitney test (p < 0.05 in bold, significant); ^d^number (%) of positive patients;^e^gingival bleeding; ^f^fisher’s exact test (p < 0.05 in bold, significant).

At acute phase of infection, leukopenia was a condition detected in both DF and DHF patients, 21 (47.7%) DHF patients and 16 (33.3%) DF patients presented leukopenia. Other clinical signs of dengue, such as abdominal pain, hypotension, and bleeding manifestations such as gingival bleeding, had been reported by 60.3%, 7.7%, and 23% of the patients, respectively. Bleeding can be associated with the reduction in platelets numbers, in fact 79% of patients with gingival bleeding were thrombocytopenic.

Once liver function can be unsettled by dengue infection, liver function tests such as total albumin, aspartate transaminase (AST), and alanine transaminase (ALT) were accessed for some patients. 19% of dengue infected patients presented low total albumin levels (< 3.5 g/dL), 70.3% high levels of AST (> 40 IU/L) and 52.6% high levels of ALT (> 55 IU/L). The average levels of total albumin, AST, and ALT were statistically different when compared DHF to DF values (Table).

Previous incidence of DENV infections was investigated and evidenced by the presence of circulating anti-DENV IgG, secondary infections were observed in 37 (77%) of DF and 41 (73%) of DHF patients (Table). DENV serotypes were identified by a serotype-specific RT-PCR assay, and we could observe that DENV-3 was the predominant DENV serotype infecting 27 patients, five patients were infected by DENV-2 and other three had DENV-1 infection.

**Fig. 1:**
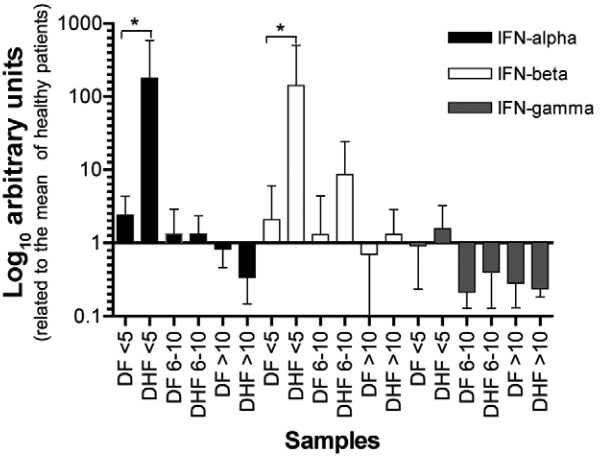
: quantitative real-time polymerase chain reaction (qRT-PCR) analysis of interferons (IFNs)α, β and y transcript level in peripheral blood mononuclear cells (PBMC) obtained from dengue virus-infected patients. 59 patients [25 dengue haemorrhagic fever (DHF) patients, 29 dengue fever (DF) patients and five healthy volunteers] had their blood samples collected and used to extract PBMC and further to obtain total RNA and cDNA. Blood samples were collected in three phases of the disease: the acute phase (< 5 days post onset of symptoms), a putatively non-viraemic phase (between six-10 days post onset of symptoms) and the convalescent phase of disease (> 10 days post onset of symptoms). The average of mRNA type-I IFN of healthy donors was used as reference for baseline of IFNs expression and is represented as 1. Data were analysed with multiple comparison Kruskal-Wallis test with Dunn’s correction. * = p < 0.05. DF n = 25 samples (< 5 days); n = 9 (six-10 days), and n = 5 (> 10 days). DHF n = 29 samples (< 5 days); n = 15 (six-10 days), and n = 15 (> 10 days).

*Immunological features of patients with dengue infection - IFN mRNA levels* - Aiming to verify the influence of DENV infection on its primary cellular targets, we conducted qPCR assays to measure the levels of the genes encoding the IFN α, β and g proteins, during the acute and convalescent phase of DENV infection. We obtained cDNA from total RNA extracted from PBMC samples of DF and DHF as templates for qPCR. In Fig. 1 it is possible to observe that the levels of IFN y are not significantly altered by DENV infection even after 10 or more days post-infection. Patients with the severe form of DHF, with up to five days of fever, presented higher levels of IFN-I (α/β) when compared to patients suffering from mild illness (DF).

**Fig. 2:**
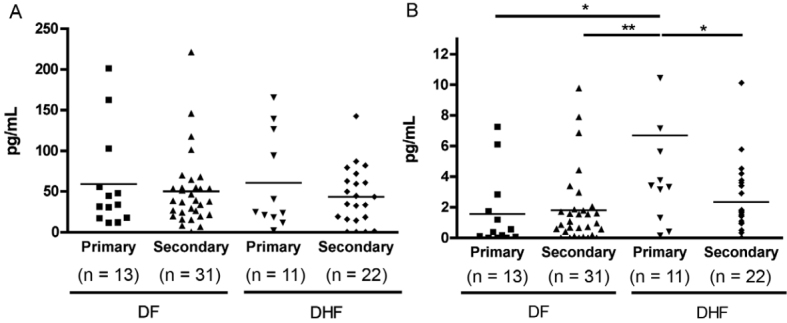
: different pattern of type I interferon (IFN) production in acute primary and secondary dengue virus infections. Serum levels of IFN- α(A) and IFN-β (B) from 44 dengue fever patients and 33 dengue haemorrhagic fever patients during acute phase of infection (< 5 days post onset of symptoms) are presented. Each dot represents one subject. All results are expressed as pg/mL. * = p < 0.05, and ** = p < 0.01 in Bonferroni’s post hoc test.

*Levels of circulating IFN-*β - IFN-I plays an essential role in antiviral defenses (Sadler & Williams 2008). To determine whether high levels of IFN-I are associated to severe dengue cases during the acute phase, both IFN-β and IFN-α were measured in serum/plasma from DF and DHF patients within five days after the onset of symptoms.Fig. 2A shows that DENV patients presented similar levels of IFN-α (primary DF: 59.05 (11.4 ~ 201.2) pg/mL; secondary DF: 50.04 (0.08 ~221.2) pg/mL; primary DHF: 60.57 (1.75 ~ 165.3) pg/mL; secondary DHF: 43.30 (0 ~ 142.6) pg/mL). These values were not statistically different. Instead, primary DHF infected patients showed the highest levels of IFN-β (6.69 (0.15 ~ 34.9) pg/mL), and it is statistically higher than primary DF (1.57 (0 ~7.24) pg/mL), secondary DF (1.80 (0 ~ 9.77) pg/mL), and secondary DHF infected patients (2.35 (0 ~ 10.1) pg/mL) (Fig. 2B).

We tried to establish association with type I IFN levels and laboratorial biochemical parameters (AST, ALT, total albumin), as well as with haematological parameters (platelets and WBC). However, type I IFN expression was not statistically correlated with any biochemical or haematological parameter.

**Fig. 3:**
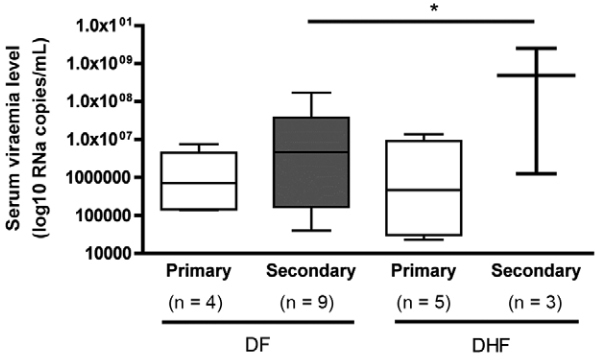
: viraemia levels in patients with primary and secondary dengue virus infections. Box-plot distribution of viraemia levels by primary and secondary dengue infection in dengue fever (DF) and dengue haemorrhagic fever (DHF) patients, in samples collected up to five days after the onset of symptoms. Lines denote median values. * = p < 0.05, in Bonferroni’s post hoc test.

*Viraemia levels in DF and DHF patients* - The same serum samples used to determine IFN-I concentrations were also used to quantify DENV viraemia levels. Results showed that 29.5% and 24.2% of the samples were DENV-RNA positive in the DF and DHF groups, respectively. Fig. 3 shows the medians of viral load in DF and DHF patients divided in primary and secondary infections. The difference observed in the virus load between secondary DF (6.66 log10 RNA copies/mL) and secondary DHF (8.68 log10 RNA copies/mL) infections was statistically significant (p < 0.05).

## DISCUSSION

In this study we compared clinical and immunological parameters in patients from northeastern of Brazil with DF and DHF. Our results show a higher incidence of DHF in adult patients (≥ 15 years old), and the development of DHF has no correlation with secondary infection, which differs to the dogma of the epidemiology for DENV infection, and this may be an evidence that the pathogenesis of dengue disease can also be influenced by virus and host factors (Pozo-Aguilar et al. 2014).

We also found that platelet counts are significantly lower in DHF than in DF patients, in agreement with the findings of Alonzo et al. (2012). Despite the efforts, the mechanism of thrombocytopenia development in DHF patients is still poorly understood. IFN-β , an important anti-virus cytokine, was described to affect platelet production in vitro (Pozner et al. 2010). Therefore, high levels of IFN-β in primary DENV infections could contribute to platelets dysfunction and coagulation disorders, highlighting a possible role for IFN-β in the immunopathogenesis of severe dengue.

Several studies highlighted the role of type I IFN in virus infection control (Diamond et al. 2000), other studies suggest that DENV infection would be associated with an increase in IFN-I expression, in vitro and in vivo (Reis et al. 2007, Becquart et al. 2010). Hernández et al. (2014) observed that, in the early stages of DENV infection, levels of IFN-α were higher in DF than in DHF patients and this early strong interferon response with better clinical outcome. Otherwise, we could not find differences in the levels of circulating IFN-α when comparing DF with DHF patients during the acute phase of infection, and this difference could be explained by the sensitivity of the methods employed to measure circulating IFN-α , or even by the virulence of the different DENV serotypes studied. Similar results were obtained by Libraty et al. (2002) regarding IFN-α in DF and DHF during secondary DENV-3 infection. We did not find a correlation between IFN-α subtype 1 ( IFN-α1) mRNA levels and the concentration of circulating IFN-α . Since the quantitative RT-PCR carried out in our study only measured IFN-α1mRNA subtype, the ELISA assay instead was able to quantify all 13 different subtypes of for circulating IFN-α, and this could had led to this discrepancy. The importance of IFN-β for the control of dengue infection was also demonstrated. Chen et al. (2013), noticed that interferon regulatory factors (IRF) 3 and 7 deficient mice were not able to control DENV replication, as consequence these animals presented a high DENV burden in different tissues. Our report presents, for the first time, the primary DHF patients presented much higher levels of circulating IFN-β than in secondary DHF or DF ones. However, other studies reported that DF and DHF patients expressed similar levels of IFN-β (Torres et al. 2015) in primary or secondary infections (Tang et al. 2010), and the reason for these differences could lie in the genetic background of different studied populations, or in the sensitivity of the tests, as well as in DENV serotypes differences.

Elevation in AST and ALT levels has been associated with dengue illness progression (Rathakrishnan et al. 2012, Ferreira et al. 2015), evidencing liver compromising during dengue infection. We also found both AST and ALT levels elevated, especially in DHF patients, however no association was found between type-I IFN circulating levels with liver transaminases.

DENV has developed different strategies to subvert the host immune response (Muñoz-Jordan et al. 2003, Aguirre et al. 2012), what would allow a more efficient virus replication evidenced by higher viraemia, what could be associated with severe dengue forms. In fact, independent studies demonstrated that DHF patients presented higher circulating levels of DENV RNA than DF patients (Vaughn et al. 2000, Libraty et al. 2002, Guilarde et al. 2008). Our results showed that secondary infected DENV patients displayed higher levels of median virus load than primary ones. However, the small number of secondary DHF patients examined, makes the strength of this observation limited.

DF patients seems to secrete suitable levels of type I IFN in primary or secondary infections, which would ensure a proper anti-viral immune response, evidenced by lower levels of circulating virus. In the other hand, DHF seems to be a condition generated by an unbalanced immune response, which compromise the virus replication control. The results of this study suggest that DENV-3 infection might elicit a strong innate immune response characterised by higher levels of IFN-β in primary DHF patients from northeast of Brazil, which could be associated with development of severe dengue on this population. The severity of infection observed in our cohort could be a consequence of the association of clinical features (low platelet counts, high viraemia, bleeding manifestation) and immunity-related factors such as high levels of IFN-β. These findings may contribute to a better understanding of dengue pathogenesis.
